# Tuberculous monoarthritis of the wrist in a patient with systemic lupus erythematosus: a case report

**DOI:** 10.1186/s13104-017-2629-2

**Published:** 2017-07-28

**Authors:** W. A. N. V. Luke, M. P. M. L. Gunathilake, Duminda Munidasa, Dilshan Munidasa, S. T. De Silva

**Affiliations:** 1 Department of Clinical Pharmacology, Faculty of Medicine, University of Kelaniya, Dalugama, Sri Lanka; 2 Professorial Medical Unit, Colombo North Teaching Hospital, Ragama, Sri Lanka; 3 Rheumatology and Rehabilitation Hospital, Ragama, Sri Lanka; 4 Colombo North Teaching Hospital, Ragama, Sri Lanka; 5 Department of Medicine, Faculty of Medicine, University of Kelaniya, Dalugama, Sri Lanka

**Keywords:** Tuberculosis, Wrist, Systemic lupus erythematosus, Case report

## Abstract

**Background:**

Unusual forms of tuberculosis are common among immune-suppressed patients, leading to challenges in diagnosis and management. We present a Sri Lankan patient with systemic lupus erythematosis, investigated for chronic wrist pain with low inflammatory markers and without systemic symptoms, who was subsequently diagnosed to have tuberculosis of the joint.

**Case presentation:**

A 31-year-old woman with systemic lupus erythematosis in remission was evaluated for chronic left wrist pain without significant examination findings on presentation. She did not have any constitutional symptoms. Basic investigations did not reveal any significant abnormalities. She was treated with increasing immunosuppression as for lupus related arthritis. Subsequently she developed a wrist effusion with high inflammatory markers, and was treated as septic arthritis. Synovial biopsy features suggested tuberculosis. The patient’s symptoms improved with surgical intervention and anti-tuberculosis treatment.

**Conclusion:**

Tuberculosis should be considered in patients with systemic arthritis with unusual symptoms. Delayed diagnosis along with continuing immunosuppression can lead to extensive tissue damage. Clinically detectable effusions should be analyzed along with synovial biopsy in order to exclude concurrent infections. Radiography of the joint has poor sensitivity to detect early joint damage, but changes may be evident early on magnetic resonance imaging, sothis should be considered in patients with unusual features.

## Background

Tuberculosis (TB) still causes a significant burden on modern healthcare all around the world, particularly in the developing world. In 2015, there were an estimated 10.4 million new (incident) TB cases worldwide, of which 5.9 million were men, 3.5 million were women and 1 million were children [1]. TB is an endemic infection in Sri Lanka. Although Sri Lanka is considered a low prevalent country in the South-East Asia region [2] around 9000 new TB cases are notified every year [3]. Nearly 60% of these are smear-positive, pulmonary TB cases [3].

Studies have shown tuberculosis to be common in patients with systemic lupus erythematosus (SLE), compared to the general population [4]. Higher cumulative dose of steroids and lupus nephritis have been shown to be important risk factors for TB [5].

Unusual presentations of tuberculosis among these patients may lead to delay in diagnosis and initiation of treatment. A high degree of suspicion is needed in assessing patients on immunosuppressive drugs and non-specific features and prolonged febrile illness. We present a patient with tuberculous arthritis of the left wrist, who was on immuno-suppressive therapy for SLE.

### Case presentation

A 31-year-old Sri Lankan woman, who was a known patient with systemic lupus erythematosus (SLE), mild persistent bronchial asthma and a history of recurrent urinary tract infections, presented with left wrist pain.

She was diagnosed with SLE 5 years previously, when she presented with photosensitivity, malar rash, inflammatory polyarthritis and supportive immunological criteria. She was treated with 1 mg/kg dose of oral prednisolone initially which was subsequently tapered to a lower dose. She was on mycophenolate mofetil with good disease control and without any organ threatening complications, when she developed wrist pain.

One year prior to the admission she developed gradually worsening pain in her left wrist, which was more towards the end of the day and worse with activity. She did not notice morning or inactivity stiffness. There was no proceeding history of trauma and other joints were not involved. She did not notice fever, oral ulcers or rashes during this period. However, she complained of fatigue. She did not notice numbness or weakness over the hand. She denied productive cough with hemoptysis or a contact history of TB.

The symptoms worsened over time with worsening disability and limitation in wrist movements. She developed intermittent short lasting bouts of fever during the course of the illness, which was treated by her general practitioner. The diagnosis was urinary tract infections in these instances based on urine full reports showing pyuria and organisms. Urine cultures were not done at these instances.

The patient was assessed by the physicians and rheumatologists from the onset of symptoms. She underwent radiography and hematological investigations, which did not reveal significant abnormalities. The pain was presumed to be due to SLE related synovitis.

She then developed persistent low-grade pyrexia for about 1 month, predominantly in the evenings with worsening joint pain. She noted joint swelling over 1 week, and the joint became excruciatingly painful with restriction of movements in all directions.

Apart from mild pallor and left wrist swelling, the general examination was unremarkable. There was clinical evidence of synovial thickening and effusion of the wrist joint, with marked tenderness and impairment of function. The power of the hand muscles was slightly diminished with wasting, without sensory impairment. System examination was unremarkable.

On admission the patient was clinically diagnosed with septic arthritis and urgent arthrotomy and wash out was done. She was started on intravenous cloxacillin and ceftriaxone after obtaining cultures. Investigations to assess the presentation and SLE disease activity were done (Table 1—investigation findings).

**Table 1:** Investigation findings

Investigation	Variable	Findings
Full blood count	Platelet count (×10^3^/μL)	312 (150–400)
	Hemoglobin (g/dL)	11.7 (12–15)
	WBC (×10^3^/μL)	7.6 (4.5–11)
	Neutrophils	45%
	Lymphocytes	51%
Erythrocyte sedimentation rate	(mm/hr)	60 (<20)
C-reactive protein	(mg/dL)	23 (<5)
Urine full report	Proteins	+
	Red cells	Nil
	White blood cells	35–40/hpf
Joint aspirate	Colour	Milky
	Appearance	Turbid
	Lymphocytes (per cumm)	100
	Polymorphs (per cumm)	Field full
	Red cells (per cumm)	400
Joint aspirate culture/synovial tissue culture	No growth	
Synovial fluid cytology	Acute inflammatory cells	
Synovial biopsy	Granulomatous inflammation, Zeihl Neelson stain positive for acid fast bacilli	
	In keeping with tuberculous synovitis	
Blood picture	Normochromic normocytic anemia with rouleux formation	
	Suggestive of chronic infection/inflammation	
TB PCR of synovial fluid	Positive for *Mycobacterium tuberculosis*	

She had normal wrist and chest radiographs, serum creatinine and liver function tests. The positivity of acid fast bacilli and granulomatous inflammation in synovial biopsy was suggestive of tuberculous arthritis. Mantoux test was negative; however this was interpreted with caution in the background of immunosuppression. Her chest radiograph did not show evidence of past or present TB. Although the presence of acid fast bacilli and granuloma on synovial biopsy were suggestive of TB, non-tuberculous mycobacteria causing infection had to be excluded.

TB PCR and cultures were subsequently available confirming *Mycobacterium tuberculosis* infection.

Intravenous cloxacillin and ceftriaxone were continued for 3 weeks until the diagnosis of TB was confirmed. The patient underwent repeated surgical drainage of the wrist 2 weeks later, as there was increasing joint swelling with expanding abscess formation. She was seen by the rheumatologist in order to optimize therapy, and was also referred for physiotherapy and occupational therapy.

The patient was educated on the diagnosis and the need of long term treatment. She was referred to the chest clinic where she was started on isoniazid, rifampicin, ethambutol and pyrazinamide along with pyridoxine. The baseline liver and renal functions were assessed prior to commencement of therapy. The treatment was continued for 2 months and a continuation phase of 10 months with isoniazid and rifampicin was planned.

## Discussion and conclusions

Skeletal involvement is seen in 1–3% of patients with TB [6]. The commonest site of involvement in bone and joint TB is the spine [7]. Weight-bearing large joints are the next to be affected, particularly hip, knee and ankle [6]. Involvement of wrist in TB is rare, though the exact prevalence is unknown. In a case series of bone and joint TB in the United Kingdom 14 out of 203 cases (6%) had wrist involvement [7].

Tuberculosis involving the wrist can be classified as cutaneous lesions, tenosynovitis, bursitis, osteomyelitis, arthritis, and tuberculous hypersensitivity reactions [8]. In a case series published in 1984, 11 cases of *Mycobacterium tuberculosis* of the hand and wrist were described [9]. This series highlighted the considerable delay between the onset of symptoms and the time of correct diagnosis. Many patients had a diagnosis of rheumatoid arthritis or nonspecific synovitis prior to the diagnosis of tuberculosis. Most patients lacked pulmonary symptoms. Only two patients had a prior history of tuberculosis and only one had significant pulmonary involvement. Most had tenosynovitis involving the flexors or extensors. Three had tenosynovitis and arthritis whereas one had only tuberculous arthritis. Two patients had carpal tunnel syndrome as a result of carpal canal involvement. Ten patients had apparent cures of tuberculosis following surgical debridement and antituberculous therapy.

A recently published study which reviewed 44 cases of wrist TB in a pediatric population highlighted the considerable delay in diagnosis despite advancement of diagnostic techniques, owing to minimal initial symptoms and rarity of the lesion. A considerable number of patients eventually developed chronic wrist pain. This study highlighted that early imaging with MRI and synovial biopsy were key to diagnosis and the most important determinant of treatment success was early initiation of antituberculous chemotherapy [10].

Our patient did not have evidence of past tuberculosis or evidence of tuberculosis at other sites. The diagnostic delay occurred as the presentation was thought to be SLE related arthritis initially, as she did not have any significant systemic symptoms or high inflammatory markers. A case series published on wrist TB in 2003 highlighted the presence of normal ESR in a significant proportion of patients [11]. Alternate diagnoses were considered as increasing immunosuppression and symptomatic treatment did not work, but there was a significant delay in diagnosis owing to the non-specific clinical features.

Our patient did not have a contact history of tuberculosis. However living in a TB endemic region, reactivation of latent tuberculosis by immunosuppression was the likely cause of the current presentation.

Diagnosis of wrist TB is often challenging. Radiographs may not show significant changes and magnetic resonance imaging is superior to radiography and CT for the diagnosis of wrist TB [9]. X-ray changes in wrist TB include bone atrophy, bone or joint destruction with discrete periostitis, and the presence of the typical spina ventosa [12, 13]. Presence of these changes in radiographs should warrant histological assessment for diagnosis of TB. Lozano et al. reported a case of TB wrist presenting with chronic wrist pain presumed to be post traumatic, but histologically evaluated due to radiological appearance of wrist bone destruction [14].

Our patient did not have significant abnormalities on serial X-rays. Magnetic resonance imaging of the wrist joint was not done due to the limited resources. MRI scanning is a good modality to demonstrate soft tissue abnormalities and provides greater detail on the extent of joint involvement, though it is not diagnostic [15]. There should be a lower threshold for advanced imaging in immune compromised patients who are more vulnerable to unusual infections.

Infections are an important cause of morbidity and mortality among SLE patients leading to premature deaths, particularly in developing countries. Tuberculosis is a leading infection among this group [16].

Studies have shown that tuberculosis is more prevalent among SLE patients [17–19] with a statistically significant increase in osteo articular TB compared to the normal population [18]. The exact prevalence of tuberculosis among SLE patients is not known; however, it varies depending on local prevalence of TB.

Uncontrolled hyperactivity of the immune system gives rise to an immuno-compromised state in SLE. Defects in cell mediated immunity caused by the disease as well as medication increases the risk of TB in the SLE patient [20]. Studies have described TB to be associated with SLE flares in some patients. It had been postulated that antibodies generated against TB may cross react with cellular antigens, which might increase flares in SLE patients and precipitate SLE in certain patients [19, 20].

As discussed above diagnosis of TB can be difficult in SLE patients due to overlapping features of the two diseases and atypical presentations [21]. Diagnosis can be made even more difficult when SLE flares coexist with tuberculosis. Pulmonary TB commonly presents without chest radiographic evidence of cavitation [22], while extra-pulmonary TB presents with fever, weight loss and skin nodules [20].

In our centre, SLE patients are not routinely screened for latent tuberculosis before starting treatment. However patients who are to be started on TNF-alpha blockers are screened for latent TB and treated. There are multiple studies evaluating the use of prophylactic anti-TB treatment in SLE patients on immunosuppressives; however, clear evidence of benefit is lacking in this regard [23].

With treatment our patient’s pain improved in severity but persisted. She was functionally disabled with disuse atrophy of small muscles of the hand.

There is limited literature on cases of wrist TB and treatment outcome. In a recent study of thirty cases of hand and wrist TB, a good outcome to anti-TB treatment was noted [24]. However, many studies have demonstrated that tuberculosis among SLE patients has high relapse rates and more complications compared to the normal population [25, 26].

In conclusion, tuberculous arthritis should be considered in the differential diagnosis in patients with chronic arthritis presenting with worsening symptoms. The onset and progression could be insidious with normal inflammatory markers, which can cause diagnostic confusion. MRI scan of the joint could facilitate early diagnosis. Though advanced imaging facilities are not freely available in resource poor settings patients with immunosuppression should be given priority, with a low threshold for imaging. Joint fluid analysis and synovial biopsy are generally helpful in the diagnosis, and should be done in patients with joint effusions whenever possible, carefully balancing the benefits and procedure related risks.

## Abbreviations

TB: tuberculosis
SLE: systemic lupus erythematosus
ATT: anti tuberculous therapy
